# Harnessing Muscle–Liver Crosstalk to Treat Nonalcoholic Steatohepatitis

**DOI:** 10.3389/fendo.2020.592373

**Published:** 2020-12-23

**Authors:** Manu V. Chakravarthy, Mohammad S. Siddiqui, Mikael F. Forsgren, Arun J. Sanyal

**Affiliations:** ^1^Axcella Health, Inc., Cambridge, MA, United States; ^2^Department of Internal Medicine and Division of Gastroenterology, Hepatology and Nutrition, Virginia Commonwealth University, Richmond, VA, United States; ^3^Department of Health, Medicine and Caring Sciences, Linköping University, Linköping, Sweden; ^4^Center for Medical Image Science and Visualization, Linköping University, Linköping, Sweden; ^5^AMRA Medical AB, Linköping, Sweden

**Keywords:** NASH, insulin resistance, lipotoxicity, myosteatosis, inflammation, skeletal muscle, adipose tissue, obesity

## Abstract

Non-alcoholic fatty liver disease (NAFLD) has reached epidemic proportions, affecting an estimated one-quarter of the world’s adult population. Multiple organ systems have been implicated in the pathophysiology of NAFLD; however, the role of skeletal muscle has until recently been largely overlooked. A growing body of evidence places skeletal muscle—via its impact on insulin resistance and systemic inflammation—and the muscle-liver axis at the center of the NAFLD pathogenic cascade. Population-based studies suggest that sarcopenia is an effect-modifier across the NAFLD spectrum in that it is tightly linked to an increased risk of non-alcoholic fatty liver, non-alcoholic steatohepatitis (NASH), and advanced liver fibrosis, all independent of obesity and insulin resistance. Longitudinal studies suggest that increases in skeletal muscle mass over time may both reduce the incidence of NAFLD and improve preexisting NAFLD. Adverse muscle composition, comprising both low muscle volume and high muscle fat infiltration (myosteatosis), is highly prevalent in patients with NAFLD. The risk of functional disability conferred by low muscle volume in NAFLD is further exacerbated by the presence of myosteatosis, which is twice as common in NAFLD as in other chronic liver diseases. Crosstalk between muscle and liver is influenced by several factors, including obesity, physical inactivity, ectopic fat deposition, oxidative stress, and proinflammatory mediators. In this perspective review, we discuss key pathophysiological processes driving sarcopenia in NAFLD: anabolic resistance, insulin resistance, metabolic inflexibility and systemic inflammation. Interventions that modify muscle quantity (mass), muscle quality (fat), and physical function by simultaneously engaging multiple targets and pathways implicated in muscle-liver crosstalk may be required to address the multifactorial pathogenesis of NAFLD/NASH and provide effective and durable therapies.

## Introduction

Tremendous progress has been made in our understanding of the mechanisms underlying non-alcoholic fatty liver disease (NAFLD) (1–3), including the identification of several molecular pathways impacting a number of cell types (hepatocytes, macrophages, stellate cells) and organ systems, ranging from the liver to adipose tissue, the gut, immune system, and kidney (4, 5). Yet, few if any of these pathways explicitly involve skeletal muscle, the principal organ responsible for glucose disposal (6) and energy homeostasis (7), key processes that can impact the core pathogenesis of a systemic metabolic disease such as NAFLD (8).

Over the past few decades, the epidemics of obesity and type 2 diabetes (T2D) have continued unabated (9). Given the known bidirectional nature of the metabolic impact of obesity/T2D and NAFLD (10), the trajectory of NAFLD has likewise increased significantly, reaching epidemic proportions, with nearly a quarter of the globe afflicted with the condition (11). Non-alcoholic steatohepatitis (NASH), the more severe form of NAFLD, with manifestations of fibroinflammatory change (12), has a global prevalence as high as 37.3% among individuals with T2D (13), contributes to increasing rates of cirrhosis (14, 15), and is rapidly emerging as the leading cause of liver transplantation (16, 17). Global prevalence rates of cirrhosis continue to increase (18) along with the proportion of cirrhotic subjects with obesity (19). Sarcopenia is common in subjects with cirrhosis, with an estimated prevalence of 40%–70%, as well as in obese individuals (20, 21). In a Korean nationwide survey, more than 12% of all patients diagnosed with NAFLD had sarcopenia independent of obesity and insulin resistance (22), and up to 30% of sarcopenic subjects without metabolic syndrome and obesity had NAFLD (23). Thus, it appears that the bidirectional muscle-liver axis could play a significant pathophysiological role across the full spectrum of chronic liver disease.

In this perspective review, we discuss three main topics: (i) current clinical evidence linking sarcopenia and NAFLD/NASH; (ii) the clinical relevance of muscle composition to physical function in NAFLD; and (iii) key pathophysiological processes and molecular mediators underpinning the muscle-liver axis in NAFLD/NASH. Among these latter processes, the review explores two key physiological concepts, anabolic resistance and metabolic inflexibility, as potential avenues for novel therapeutic strategies to address the complex and multifactorial pathogenesis of NAFLD/NASH.

## Sarcopenia—Definitions and Measurements

### Definitions

The term “sarcopenia” was first introduced by Irwin Rosenberg in 1989 to describe the age-related decline in muscle mass among the elderly (24). Muscle mass accounts for ~45% of body mass, and once people reach 50 years of age, they lose ~1%–2% of their muscle mass per year (25). The European Working Group on Sarcopenia in Older People (EWGSOP) defines sarcopenia as generalized and progressive loss of three parameters: (i) muscle strength, (ii) muscle quantity/quality, and (iii) physical performance (26). Loss of muscle quality, for example as a result of myosteatosis, has also been directly linked to low physical function, poor clinical outcomes, and mortality (27).

**Figure 1:**
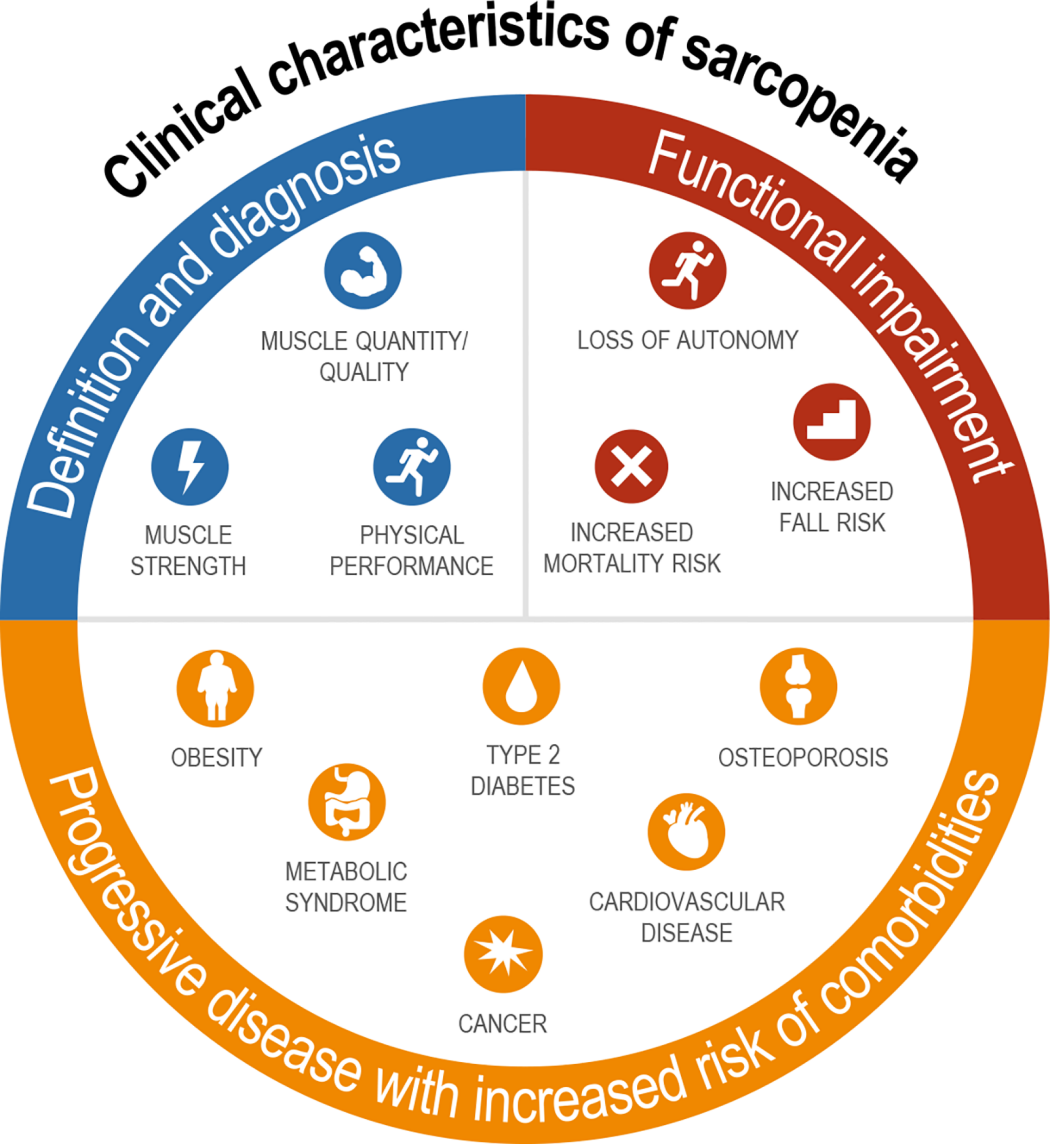
Definition, sequelae, and related comorbidities of sarcopenia. Diagnosis includes assessment of both muscle mass and strength with functional impairments seen across multiple domains; sarcopenia is associated with nearly every major chronic disease.

Sarcopenia is clinically meaningful as it results in functional impairment with loss of strength, disability, frailty, loss of autonomy, and increased risk of falls and mortality, and therefore fundamentally affects how an individual feels and functions (**Figure 1**) (28–30). Although sarcopenia was once regarded as part of normal aging (31), nowadays, it is increasingly recognized as a progressive disease that is associated with increased risk of several common chronic metabolic disorders, including obesity, type 2 diabetes, metabolic syndrome, osteoporosis, cardiovascular disease, and cancer (**Figure 1**) (32–37). Sarcopenia is also acknowledged as a common complication and mortality risk factor in patients with cirrhosis and end-stage liver disease (ESLD) (38, 39). However, there are limited data directly linking sarcopenia to outcomes in NASH.

### Measurements

The Foundation for the National Institutes of Health Sarcopenia Project, comprising a pooled sample of 26,625 participants [57% women, mean age in men 75.2 (± 6.1 standard deviation) and in women 78.6 (± 5.9) years], recommended the following cutoff points for weakness and low lean mass: handgrip strength <26 kg for men and <16 kg for women, and appendicular lean body mass [measured by dual-energy X-ray absorptiometry (DXA) and adjusted for body mass index (BMI)] <0.789 for men and <0.512 for women (40). Recommendations from the EWGSOP (26) to assess for evidence of sarcopenia include strength assessments with the use of handgrip strength (<27 kg for men, <16 kg for women) and chair stand (>15 s for 5 rises); to confirm sarcopenia by detection of low muscle quantity and quality, DXA is advised in clinical practice, and DXA, bioelectrical impedance analysis, computerized tomography (CT), or magnetic resonance imaging (MRI) in research studies with appendicular skeletal muscle mass <20 kg (<7.0 kg/m^2^) for men and <15 kg (<5.5 kg/m^2^) for women. To determine severity of sarcopenia, recommendations include physical performance measures of gait speed (≤0.8 m/s), short performance physical battery (≤8 point score), timed-up-and-go test (≥20 s), and 400-m walk test (non-completion or ≥6 min for completion). While these are the current EWGSOP recommendations, it is important to acknowledge that some of these assessments (e.g., gait speed, 400-m walk) may also depend on cardiopulmonary fitness. In addition, assessment of strength by hand grip and chair stand as proposed addresses two distinct muscle groups, and consequently, could impact prognosis differently. Thus, additional studies are likely needed to further delineate the contribution of cardiopulmonary fitness to these tests designed to measure sarcopenia *per se*, as well as when to use one strength test or the other.

## Clinical Evidence Linking Sarcopenia and Non-Alcoholic Fatty Liver Disease/Non-Alcoholic Steatohepatitis

### Meta-Analyses

Four large meta-analyses (*N* ranging from 3,000 to ~30,000) estimated that individuals with sarcopenia were at ~1.3- to 1.5-fold increased risk of NAFLD compared to those without sarcopenia (41–44). In the few studies that examined the association between sarcopenia and NASH/fibrosis, the odds ratio (OR) for NASH was ~2.4, and for advanced liver fibrosis the OR ranged from ~1.6 to ~2.4 across the various studies (41, 42, 44). Conversely, skeletal muscle index (SMI) (skeletal muscle mass divided by height squared or weight) in NAFLD patients was ~1.8-fold lower (95% CI: 1.15–2.39) than that in healthy controls (42). However, there was generally high heterogeneity among the studies (I^2^ range 61%–98%).

### Population-Based Studies

High-quality population studies have emerged over recent years to explore the relationship between sarcopenia and the presence and severity of NAFLD (25, 45). In studies conducted in Chinese and European individuals, SMI was inversely associated (OR 0.1–0.48), and intramuscular fat was positively associated (OR ~2–10), with NAFLD (46, 47). Several cohort and cross-sectional studies have indicated that SMI may be closely associated with the incidence of NAFLD (48–51), and that a low SMI is associated with metabolic dysregulation and NAFLD progression (52, 53). Among patients with NAFLD, the presence of sarcopenia was associated with a 2.5-fold increase in the risk of NASH (52). Advanced liver fibrosis was seen more often in those with sarcopenia (7.8%) compared to those without (1.6%), and sarcopenia was associated with advanced liver fibrosis (OR 1.8), independent of other metabolic risk factors (54).

**Figure 2:**
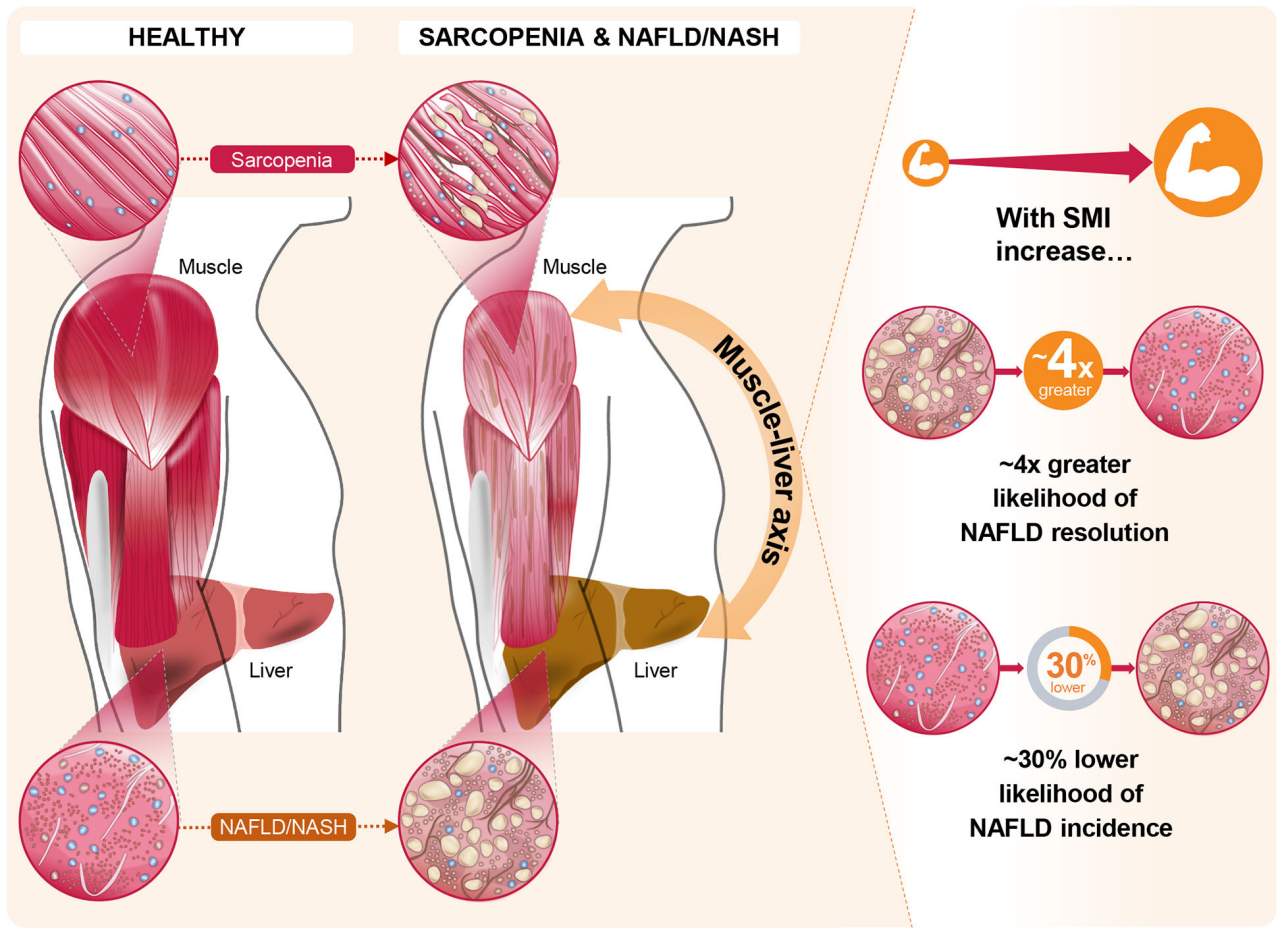
The role of the muscle-liver axis in sarcopenia and non-alcoholic fatty liver disease (NAFLD)/non-alcoholic steatohepatitis (NASH). Recent evidence in the context of NAFLD and NASH, such as that from Kim et al (49). offers compelling associations between changes in skeletal muscle index (SMI) and both NAFLD incidence and resolution of existing NAFLD. In this longitudinal study, these marked associations persisted for the highest tertile of SMI change over 1 year, relative to the lowest tertile, even after full adjustments for multiple covariates including baseline SMI.

The impact of skeletal muscle mass and its changes over time on the development of incident NAFLD or the resolution of baseline NAFLD were studied in a cohort of 12,624 subjects without baseline NAFLD and 2,943 subjects with baseline NAFLD (49). In this study, NAFLD was assessed by hepatic steatosis index, and SMI was estimated by bioimpedance analysis. Over a 7-year follow-up period, ~15% of the total population without baseline NAFLD developed NAFLD. Increased SMI was associated with reduced incidence of NAFLD [adjusted hazard ratio (AHR): 0.84 (95% CI: 0.79–0.90) per percent increase in SMI over 1 year]. Similarly, participants in the highest tertile of change in SMI over 1 year (compared with the lowest tertile) had both a lower likelihood of incident NAFLD [AHR: 0.69 (95% CI: 0.59–0.82)] and a higher likelihood of resolution of baseline NAFLD [AHR: 4.17 (95% CI: 1.90–6.17)] even after adjustment for multiple covariates, including baseline SMI (**Figure 2**) (49). Subjects in the highest tertile of change in SMI over 1 year also showed the greatest reductions in BMI, alanine aminotransferase and aspartate aminotransferase, fasting glucose, homeostatic model assessment of insulin resistance, lipid parameters, and hepatic steatosis index score (49). These findings suggest that increases in skeletal muscle mass over time may slow, halt, or reverse NAFLD development and facilitate resolution of existing NAFLD.

In the prospective, observational Korean Sarcopenic Obesity cohort study of 452 apparently healthy adults (25), individuals with lower skeletal muscle mass (as measured by DXA to estimate SMI) exhibited increased risk of NAFLD (defined by the liver attenuation index measured using abdominal CT). In a multiple logistic regression analysis, the OR for NAFLD was 5.16 (95% CI: 1.63–16.33) in the lowest quartile of SMI compared to the highest quartile after adjusting for age and gender; this association remained independent of insulin resistance (25). A subsequent population-based nationwide survey (Korea National Health and Nutrition Examination Survey 2008–2011) corroborated these findings by demonstrating that sarcopenia was associated with NAFLD independent of obesity and insulin resistance (23). There was also a strong graded response with disease severity for NASH and fibrosis stage, both independent of obesity (52, 55).

### A Few Limitations of Current Clinical Evidence

In most of these studies, both skeletal muscle mass and liver fat assessments were based on a variety of methods without a uniform reference standard. The most commonly used techniques were bioelectrical impedance analysis and DXA to estimate muscle mass. Previous studies have shown that both techniques are fraught with accuracy, sensitivity, and reproducibility issues (56–60). Cross sectional imaging (e.g., by CT or MRI) are considered to be gold standards, but do not lend themselves readily to use in large trial settings (61). In addition, while CT is sensitive for detecting moderate to advanced hepatic steatosis, it has limited diagnostic performance to assess mild steatosis (62). In almost all of the population studies, there was limited to no information on function (strength or muscle quality). Most studies used surrogate indices for NASH diagnosis, and only 2 studies used liver biopsy (52, 55). There were also large differences in the populations studied, with Asian populations predominating, and limited evidence from other ethnic groups. Although adjustment for common confounders such as age and gender was usually performed, there was less frequent adjustment for other known confounders such as inflammation and physical activity. Finally, most studies were cross-sectional in nature, complicating attempts to establish a cause-effect relationship.

## Muscle Composition and Physical Function in Non-Alcoholic Fatty Liver Disease

Muscle composition is a major determinant of global muscle metabolic function, strength, and physical performance (63). Detailed measurement of muscle composition includes not only muscle mass, but also quantification of fat-free muscle volume and muscle fat infiltration (myosteatosis) (64). Myosteatosis has been linked to metabolic, functional, and clinical outcomes (65), a higher risk for cirrhosis-related complications such as hepatic encephalopathy (66), and overall mortality in patients with cirrhosis (67). The UK-Biobank (UKBB), a large and detailed prospective study following 500,000 healthy volunteers in the UK, gathered extensive datasets based on physical examinations, blood and urine samples, genetic profiles, patient health-related quality-of-life questionnaires, functional performance measures such as handgrip strength, walking pace, stair climbing, and falls, and health outcomes such as hospitalization and death (64, 68). Data from approximately 10,000 UKBB participants demonstrated that muscle composition (based on fat-free muscle volume and myosteatosis by water-fat separated neck-to-knee MRI), even after adjustment for age, gender and BMI, was more strongly associated with physical function, activities of daily living, and hospitalization than muscle volume alone, enabling an objective and improved definition of sarcopenia that is unaffected by body size (64).

**Figure 3:**
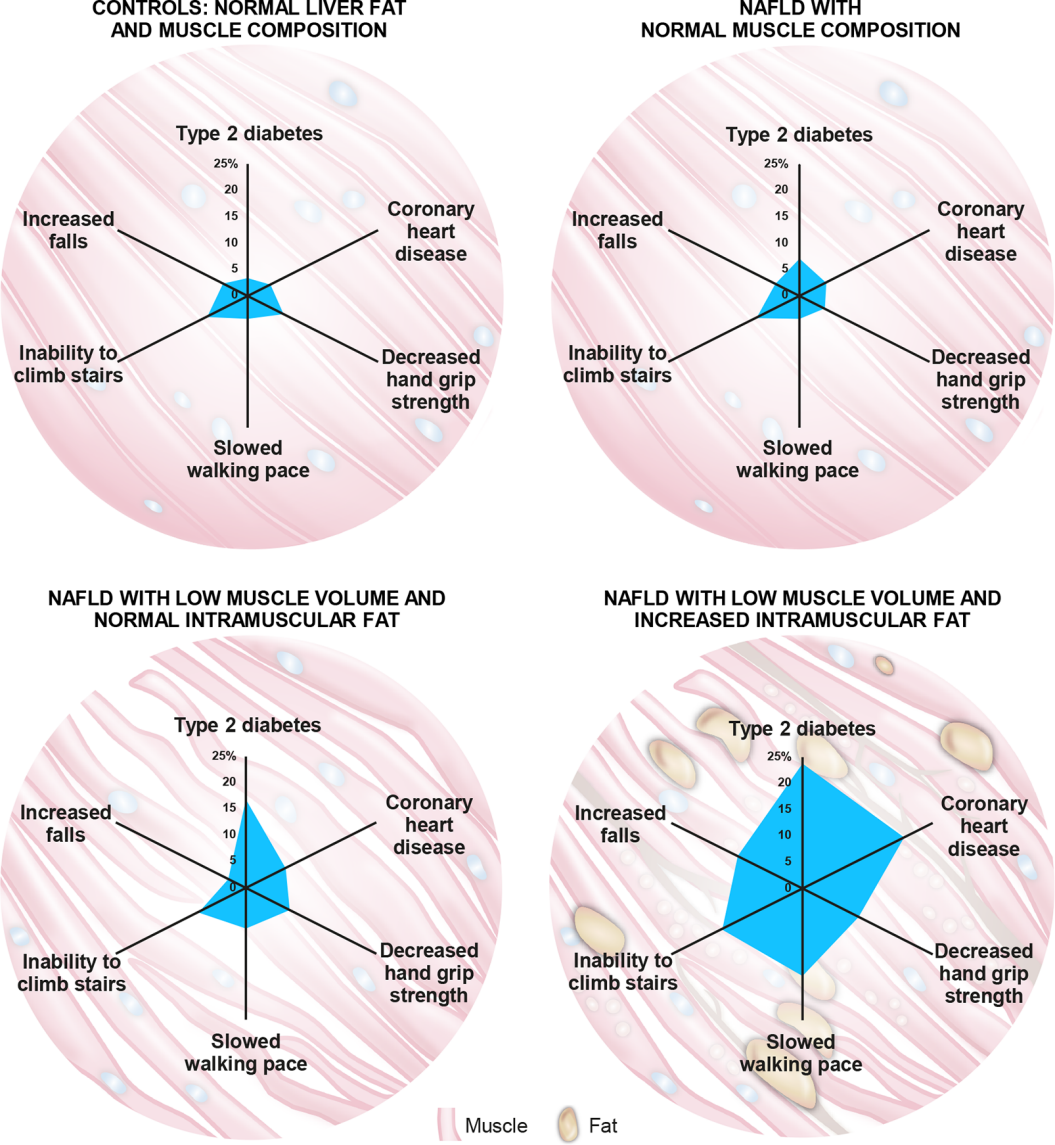
The role of muscle composition in non-alcoholic fatty liver disease (NAFLD) and related comorbidities. A recent analysis of the UK-BioBank (UKBB) resource by Linge et al (69). revealed that participants with NAFLD and normal muscle composition had generally similar metabolic and functional characteristics to those with normal liver and muscle composition. Interestingly, participants with NAFLD combined with adverse muscle composition (AMC), defined as the presence of both low muscle volume (i.e., <25th percentile of the UKBB population) and high muscle fat infiltration (i.e., >75th percentile of the UKBB population), exhibited a larger “footprint” (higher prevalence) of relevant comorbidities and functional impairment when compared with the other groups evaluated. Numbers on axes represent prevalence (%) of each indicated comorbidity/functional impairment.

Recent evidence shows that muscle composition also plays a significant role in NAFLD and related comorbidities. The UKBB resource was investigated for the impact of MRI-measured adverse muscle composition (AMC), defined as the presence of low muscle volume (i.e., <25^th^ percentile of the UKBB population) in conjunction with high muscle fat infiltration (i.e., >75^th^ percentile of the UKBB population) in 1,204 participants (women: 46.4%; mean age: 62.9 years; mean BMI: 30.1 kg/m^2^) with NAFLD (defined as MRI-proton density fat fraction, PDFF >5%), and low alcohol consumption (less than 14 and 21 units/week for females and males, respectively) and those without NAFLD (n = 4,122; MRI-PDFF </=5% with low alcohol consumption). In this study, muscle fat was significantly elevated in those with NAFLD vs. those without (8.03 ± 2.08% vs. 7.21 ± 1.82%; p < 0.001), and muscle fat infiltration above the 75th percentile was present in 37.8% of those 1,204 individuals with NAFLD (69). AMC was found to be highly prevalent, with 14.0% of the participants with NAFLD having both low muscle volume and high muscle fat (69). In NAFLD subjects, those with AMC as compared to those with low muscle volume alone had a higher prevalence of T2D (23.7% vs. 16.8%) and coronary heart disease (19.5% vs. 7.6%), as well as poor activities and function of daily living, as indicated by higher prevalence of decreased handgrip strength (10.7% vs. 8.4%), slow walking pace (16.6% vs. 7.6%), inability to climb stairs (15.4% vs. 9.2%), and more than one fall in the preceding year (12.4% vs. 3.4%) (69) (**Figure 3**). Interestingly, NAFLD participants presenting with normal muscle composition had similar background metabolic and functional risk as the control (low liver fat and alcohol consumption) population (**Figure 3**), with the exception of a higher T2D prevalence (69).

The advent of high-precision volumetric measurements in tomographic images such as MRI and CT has also allowed detailed quantification of myosteatosis in those with NAFLD. In a general cohort of 6,021 participants, median muscle fat infiltration was 7.19% (IQR: 6.18–8.42) (70). In the cohort with NAFLD, muscle fat infiltration in those with normal muscle composition was 6.78 ± 1.05%, similar to that of the general population; however, in NAFLD subjects with adverse muscle composition, muscle fat content was 10.10± 2.11% (p < 0.001) (69). The observation of higher myosteatosis in NAFLD subjects is not just restricted to the quadriceps. MRI-based muscle fat infiltration of the spinal erector muscle group (iliocostalis, longissimus, and spinalis) showed an absolute increase of 2.3 percentage points in subjects with NAFLD (10.9%) as compared to those with other chronic liver diseases (8.6%), and this number increased further with liver fibrosis stage [absolute increase of 5.0 percentage points in those with F3/F4 (14.9%) vs. F0 to F2 fibrosis (9.9%)] (71). Using a CT-based evaluation, another group (72) independently demonstrated higher fat accumulation within the psoas muscle (indicated by 15%–20% lower muscle density) in subjects with NASH with or without fibrosis compared to those with only fatty liver (NAFL); there was no graded response of muscle fat accumulation with fibrosis stage, as NASH subjects with either fibrosis F0/F1 or F2–F4 had similarly decreased muscle density. In a multivariate analysis, only relative muscle density and alanine transaminase emerged as independent predictors of NASH (72). Cumulatively, although these provocative results seem to suggest that myosteatosis by itself could be both a diagnostic and prognostic marker in NAFLD, additional prospective studies would be needed to confirm these initial observations. It also remains to be determined if there is a differential metabolic response to muscle composition changes within peripheral (thigh) and central (spinal erector) muscle groups.

Taken together, abnormal muscle composition in NAFLD, namely low mass with increased myosteatosis, was independently associated with low physical function and was largely under-diagnosed (69). These findings suggest that highly vulnerable populations may not be detected using current sarcopenia measurement tools and that more advanced imaging may help to identify those at risk of impaired physical function (64). Although low muscle volume alone confers greater risk of functional disability in those with NAFLD as compared to age-, gender-, and BMI-matched controls, the current evidence suggests that the presence of both low muscle volume and high muscle fat may amplify this risk (**Figure 3**). Thus, assessing muscle composition in NAFLD using a non-ionizing radiation technique such as MRI that can reliably and reproducibly assess longitudinal changes in muscle composition over time (test-retest repeatability coefficient was 0.53 percentage points for muscle fat infiltration) (73), enables its utilization in clinical trial settings to more robustly characterize both pathophysiology and prognosis: the ability to differentiate between vulnerable and normal sub-groups would aid in selecting a more appropriate (and homogenous) NAFLD population for clinical trials, and in tailoring appropriate therapeutic interventions. The availability of objective and highly reliable biomarkers of overall body composition, including muscle quantity and quality (73), would also enable tracking of muscle health, sarcopenic processes, and comorbidities at a much earlier stage and before onset of physical dysfunction. With proper adjustment for body size, these biomarkers avoid known confounding factors unrelated to muscle health or patient fitness (70).

## Key Mechanisms and Molecular Factors at the Nexus of Non-Alcoholic Fatty Liver Disease/Non-Alcoholic Steatohepatitis And Sarcopenia

### Muscle-Liver Axis

**Figure 4:**
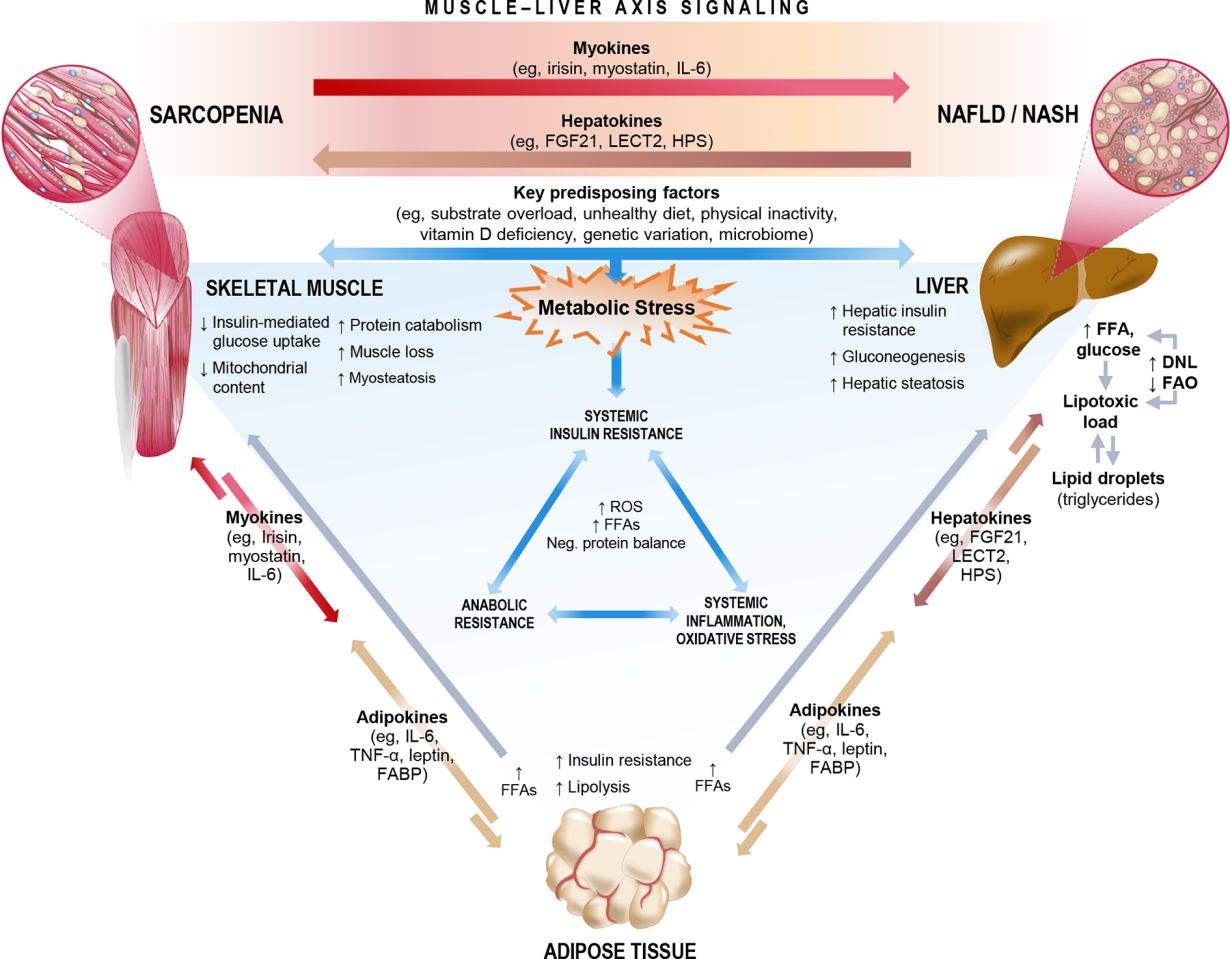
Key mechanisms and molecular signals that link sarcopenia and non-alcoholic fatty liver disease (NAFLD)/non-alcoholic steatohepatitis (NASH). The complex interorgan crosstalk between liver and muscle likely shares a number of key underlying mechanisms, many of which relate both sarcopenia and NAFLD/NASH to metabolic stress, cascading to biochemical pathways that impact systemic insulin resistance, inflammation/oxidative stress, and anabolic resistance. Among these mechanisms are a number of existing or emergent predisposing factors and the release of multidirectional molecular signals consisting of myokines, hepatokines, and adipokines. In the context of sarcopenia, skeletal muscle could exert dysregulated influence on the muscle-liver axis to potentially play a causative role in NAFLD incidence or progression. DNL, *de novo* lipogenesis; FABP, fatty acid-binding protein; FAO, fatty acid oxidation; FFA, free fatty acid; FGF21, fibroblast growth factor 21; HPS, hepassocin; IL-6, interleukin-6; LECT2, leukocyte cell-derived chemotaxin-2; ROS, reactive oxygen species; TNF-α, tumor necrosis factor-alpha.

The relationship between ESLD and sarcopenia is well established (66, 74, 75). Recent studies, as summarized in Section 2, also highlight an important association between sarcopenia and NAFLD, even among patients who have not yet progressed to ESLD, highlighting the central role of the muscle-liver axis. NAFLD is considered both as a precursor of the metabolic syndrome (76) and as the hepatic manifestation of the metabolic syndrome (77), and therefore likely shares common key mechanisms that link sarcopenia and the metabolic syndrome. Interorgan crosstalk between muscle and liver is influenced by several factors, including underlying obesity, low physical activity, vitamin D deficiency, oxidative stress, a proinflammatory milieu, and insulin resistance. Lipotoxicity induced by fatty acid (FA) overload can also lead to ectopic fat deposition in multiple organs, including liver (hepatic steatosis) and skeletal muscle (myosteatosis), and is likely mediated by hepatokines and myokines (**Figure 4**) (78, 79). In this sense, skeletal muscle could play a causative role in NAFLD through dysregulated secretion of various myokines against the background of sarcopenia. **Figure 4** summarizes the proposed mechanisms linking sarcopenia and NAFLD/NASH.

### Key Mechanisms

#### Anabolic Resistance

Dysregulated nitrogen homeostasis underpins impaired hepatic metabolism (80), whereby an imbalance between muscle protein synthesis and muscle protein breakdown ultimately contributes to the decreased muscle mass that accompanies liver disease (81). Protein synthesis in skeletal muscle is activated by anabolic factors such as amino acids (AAs), hormones (insulin, growth hormone, and insulin-like growth factor 1), and mechanical stimulus (muscle contraction) (82). In contrast, protein catabolism is activated by energy deficiency and systemic inflammatory processes (83). Thus, maintenance of muscle mass requires that skeletal muscles are responsive to AA provision, hormonal stimulation, and/or muscle contraction. Consequently, anabolic resistance, the inability of an anabolic stimulus to provide adequate stimulation of muscle protein synthesis, could constitute a key unifying mechanism for the muscle mass loss commonly seen in the setting of NAFLD (**Figure 4**).

In the fasted state, protein balance is negative since protein synthesis falls below protein catabolism. In contrast, after a meal, protein balance normally becomes positive as protein synthesis increases and proteolysis diminishes, particularly if the meal is high in protein. In the setting of disease (such as NASH, cirrhosis, and post-liver transplantation) or aging, the ability to synthesize protein in response to various nutritional factors (dietary protein, AAs, and insulin) appears to be blunted (82). The result is a negative protein balance with a steady and progressive decline in protein stores. Furthermore, optimal activation of protein synthesis after a meal also depends on the availability of specific signaling AAs (84, 85) such as leucine [via its potent activation of mammalian target of rapamycin (mTOR) complex 1] (86) and arginine (via its synthesis of nitric oxide to increase muscle blood flow for substrate supply) (87). Taken together, anabolic resistance may be attributed to inadequate AA availability/delivery, insulin resistance, and/or systemic inflammation, all of which may be further exacerbated in obese older adults.

By taking a rational approach to provide an optimal AA composition, these defects could potentially be overcome. As one example, in a study of prefrail (but not malnourished) subjects with compensated cirrhosis (Child-Pugh Class A and B) largely due to NASH, a defined composition of 8 AAs (leucine, isoleucine, valine, histidine, lysine, threonine, ornithine, and aspartate) in specific ratios (AXA1665; Axcella Health Inc., Cambridge, MA) resulted in leaner body composition (higher % lean mass with lower % body fat mass) coupled with significant improvement in the Liver Frailty Index, a composite physical function assessment of handgrip strength, timed chair stands, and balance (88). Anabolic resistance may also be overcome with exercise. In an individualized web-based exercise program that combined endurance and strength training with bidirectional feedback carried out over 8 weeks in a prescribed sequence to stimulate muscular strength in patients with histologically confirmed NASH, improvements in markers of steatosis (decreased fatty liver index), fibrogenesis (decreased ProC3) and fibrinolysis (increased C4M2) were demonstrated (89). Together, such interventional studies suggest that modulation of muscle physiology to overcome anabolic resistance could be a core pathway to impact body composition, physical function, and hepatic fibrosis remodeling in subjects with NASH.

#### Insulin Resistance

Skeletal muscle is the principal organ in energy metabolism and is responsible for insulin-mediated glucose uptake, which occurs *via* glucose transporter 4. Animal models with muscle-specific glucose transporter 4 knockouts develop severe insulin resistance (90). In addition to stimulating uptake of glucose, insulin also enhances protein synthesis, inhibits proteolysis, and stimulates AA transport in skeletal muscle (91, 92). Insulin also increases the supply of nutrients to muscles through its vasodilatory properties and, consequently, plays an important role in the physiological coupling between hemodynamic and metabolic homeostasis (93). Insulin, *via* its activation of p38 mitogen-activated protein kinase (MAPK) and mTOR, stimulates mRNA translation of genes responsible for muscle proliferation and hypertrophy (94). These effects of insulin on muscle are blunted in the state of insulin resistance, and could lead to anabolic resistance, characterized by reduced protein synthesis and reduced insulin-mediated suppression of protein catabolism (95–97). Impaired insulin-stimulated glucose uptake into muscle leads to further deterioration of whole-body glucose homeostasis and worsening sarcopenia. Thus, insulin resistance, which is also an underlying driver of NAFLD pathogenesis (98, 99), directly links NAFLD and sarcopenia (**Figure 4**).

In NAFLD, weight gain is associated with visceral adipose tissue expansion and infiltration of adipose tissue by macrophages and adipose tissue inflammation (100). This local inflammatory milieu promotes development of insulin resistance at the level of adipose tissue (101). Furthermore, insulin and other effectors of skeletal muscle anabolism (i.e., resistance exercise and essential AAs) are less effective at inducing skeletal muscle protein synthesis in the presence of increased adiposity (102, 103). Thus, loss of muscle mass can lead to significant whole-body metabolic disturbances that include decline in basal metabolic rate, and loss of mitochondrial volume, density, and oxidative capacity, with further exacerbation of muscle loss (**Figure 4**) (104, 105). Conversely, improving skeletal muscle oxidative capacity as exemplified by exercise training (both resistance and aerobic training) has been shown to significantly reduce intrahepatic fat content independent of weight loss in subjects with fatty liver and type 2 diabetes (106, 107).

Normal energy metabolism is characterized by periodic shifts between glucose and FA oxidation depending on fuel availability (108). Skeletal muscle is a major contributor to whole-body energy expenditure and thus is a key organ in energy homeostasis (7). The ability to preferentially use the appropriate fuel substrates (i.e., FAs during fasting and carbohydrate in fed states) for energy generation is referred to as *metabolic flexibility* (109). In the normal fasted state, serum insulin levels decrease, thereby releasing insulin-mediated suppression of lipolysis of adipose tissue. This results in a steady supply of FAs to be used as the major fuel source during fasting (110). In the postprandial state, meal-induced insulin secretion facilitates the transport of glucose into intracellular compartments, where it is used as the preferred fuel source. When the body is unable to preferentially utilize the appropriate fuel sources at the appropriate energy state, the result is *metabolic inflexibility*, which is associated with weight gain, diabetes, and NASH (1, 111–113).

**Figure 5:**
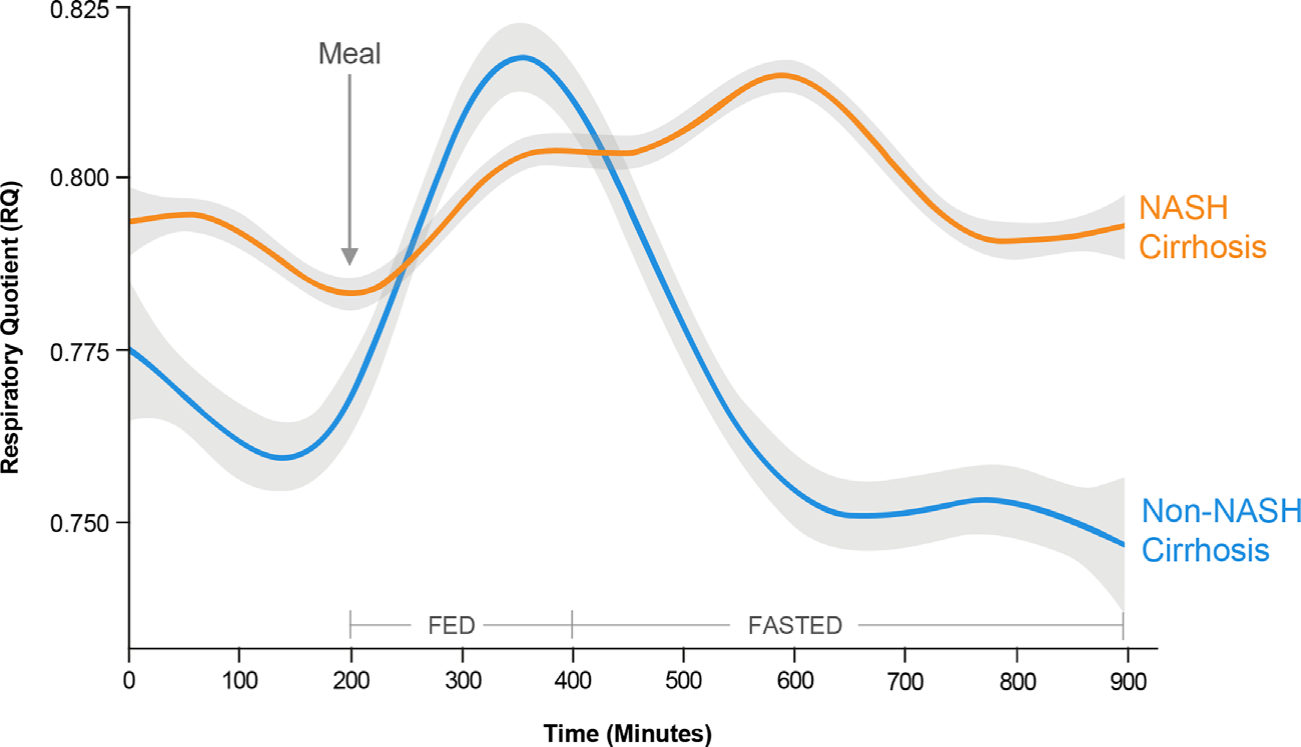
Metabolic inflexibility in non-alcoholic steatohepatitis (NASH). Continuous respiratory quotient (RQ) evaluations in a whole-room calorimetry study revealed less efficient biofuel switching, i.e., metabolic inflexibility, among subjects with NASH cirrhosis compared with non-NASH cirrhosis, manifesting as a delayed time to peak RQ in a fed state, and an inability to switch to lower RQ in a fasted state. These findings reflect an impaired ability of skeletal muscle to utilize fatty acids for oxidation in the fasted state in subjects with NASH cirrhosis. [Adapted from Siddiqui et al (116). Copyright 2019, with permission from Wiley.].

In a metabolically inflexible state, normal pulsatile insulin release in response to the level of satiety is impaired such that basal insulin levels remain high even in the fasted state (109). Despite hyperinsulinemia, insulin resistance at the level of adipose tissue results in adipose tissue lipolysis, and increased generation of circulating FAs that are not utilized for oxidation (114). Thus, a hallmark of metabolic inflexibility is the impaired ability of skeletal muscle both to oxidize FAs in the fasted state and to switch to carbohydrate utilization in the fed or insulin stimulated state (115). This concept was recently demonstrated using whole-room calorimetry with continuous 18-hour monitoring in a cohort of patients undergoing liver transplantation for NASH- and non-NASH-related cirrhosis (116). The cellular rate of carbon dioxide production relative to oxygen consumption [respiratory quotient (RQ)] is used to quantify whole-body fuel utilization: an RQ value of 0.7 is indicative of pure FA oxidation, whereas an RQ of 1.0 is indicative of pure carbohydrate oxidation. After a standardized meal, patients undergoing transplantation for NASH-related cirrhosis took longer to switch to carbohydrate metabolism than those with non-NASH-related cirrhosis (514 vs. 430 min; *p* = 0.03), indicating less efficient biofuel switching in the fed state (**Figure 5**). Patients from both cohorts had similar peak RQ values, indicating that although it took patients with NASH-related cirrhosis longer to switch to carbohydrate metabolism, they were still able to reach the same magnitude of carbohydrate metabolism as patients with non-NASH-related cirrhosis. Similarly, in the fasted state, patients with NASH-related cirrhosis took longer to reach the lowest RQ, again reflecting less efficient switching of biofuel utilization toward fat oxidation (**Figure 5**). Finally, patients with NASH-related cirrhosis had significantly higher RQ values even during prolonged fasting, indicating continued reliance on carbohydrates, even under low carbohydrate conditions (**Figure 5**). Due to the impaired ability to oxidize FAs in this metabolically inflexible state, excess FAs are stored within the muscle leading to accumulation of intramyocellular lipid (myosteatosis). This relationship was also confirmed in the above-mentioned study, where an inverse relationship between myosteatosis and metabolic flexibility was demonstrated (116). Furthermore, myosteatosis has been associated with reduced muscle protein synthesis, linking insulin resistance to sarcopenia (117).

#### Systemic Inflammation

Recent studies recognize both NAFLD and obesity as subclinical inflammatory states (118, 119). Indeed, metabolic inflammation emanating from the fatty liver is postulated as a key driver of downstream cellular dysfunction, cell death, and deleterious remodeling within various body tissues, possibly including skeletal muscle (120). In obesity, increased adipose tissue secretes adipokines and other proinflammatory cytokines (**Figure 4**), which promote infiltration of inflammatory cells, including macrophages (100). The infiltrating macrophages change their phenotype from M2 to M1 and release proinflammatory cytokines such as interleukin (IL)-6, tumor necrosis factor (TNF)-α, and IL-1β (121, 122). These cytokines negatively impact skeletal muscle by upregulating proteasomal decay of filament proteins and promoting apoptosis (123). Incremental release of IL-6 under normal physiological conditions (e.g., muscle contractile activity) improves insulin signaling by enhancing glucose uptake and increasing FA oxidation in myocytes *via* phosphoinositide 3-kinase (PI3K) and AMP-activated protein kinase (AMPK) (124), while also inducing anti-inflammatory cytokines (i.e., IL-10) (125). However, in chronic inflammatory states such as those that may occur in obesity and NAFLD, IL-6 acts as a proinflammatory cytokine, reducing myogenesis by inhibiting insulin-like growth factor (IGF)-1 activity *via* activation of suppressor of cytokine signaling-3 (SOCS-3) (126, 127).

### Other Key Molecular Factors

Other reviews have extensively covered molecular mediators underlying both sarcopenia and NAFLD (78, 128, 129). Here, we focus primarily on three key factors that can significantly influence muscle-liver crosstalk by modulating glucose homeostasis and insulin resistance to impact NAFLD pathogenesis and disease progression.

#### Myostatin

Myostatin is a well-established myokine that plays a central role in inhibiting skeletal muscle growth and mass (130). In patients with ESLD, four-fold elevated serum myostatin levels are reported (131). Myostatin has both local and endocrine effects that can link sarcopenia and NAFLD *via* a complex signal transduction process involving downregulation of genes controlling myogenesis and muscle protein synthesis, while simultaneously activating proteasome–ubiquitin ligases (132). Metabolically, myostatin regulates glucose disposal and adiposity, including increased browning of adipose tissue (133). Deletion of myostatin in mouse models produces dramatic improvements in insulin sensitivity and glucose uptake, and a reduction in adiposity (134). Inactivation or absence of functional myostatin increased lipolysis and FA oxidation in peripheral tissues, increased muscle mass (135, 136), and ameliorated fatty liver in mice (137). Although the exact mechanism is not entirely clear, a myostatin receptor has been reported on hepatic stellate cells (138, 139). It has recently been demonstrated that myostatin reduced human stellate cell proliferation, induced cell migration, and increased expression of procollagen type 1, tissue inhibitor of metalloproteinase-1, and transforming growth factor-β1 (139), further implicating myostatin as a key molecular mediator of muscle-liver crosstalk.

#### Irisin

Insulin resistance also impacts the myokine profile of skeletal muscle, promoting impaired skeletal muscle growth and proliferation. Irisin, a myokine, acts on skeletal muscle, resulting in increased energy expenditure and oxidative metabolism *via* regulation of cellular energetics (140, 141) and is a critical mediator of hepatic glucose and lipid metabolism (142). Irisin expression in skeletal muscle is reduced in obesity and is related to insulin sensitivity (143). Irisin improves glucose homeostasis, increases adipocyte energy expenditure, and modulates the expression of enzymes that inhibit lipid accumulation and reduces weight (144, 145). In adipocytes, irisin promotes differentiation of white adipose tissue to brown adipocytes, thereby underscoring the beneficial pleiotropic effects of irisin in improving adipocyte metabolism (140). Thus, it is plausible that decreased skeletal muscle could be a causative factor of NAFLD incidence due to reduced secretion of various salutary myokines.

#### Vitamin D

Vitamin D deficiency has been implicated as a potential contributor to both muscle- and liver-related metabolic derangements (146). Vitamin D regulates expression of insulin receptors in pancreatic β-cells (147, 148) and peripheral target tissues (149). Vitamin D receptor is expressed within the liver (150) and may mediate hepatic injury *via* modulation of systemic inflammation and oxidative stress (151). Clinically, patients with NAFLD have lower levels of vitamin D (152). Furthermore, vitamin D receptor expression on hepatocytes inversely correlates with severity of liver disease, while accounting for traditional metabolic risk factors (153). In the Longitudinal Aging Study Amsterdam, vitamin D deficiency (25-hydroxyvitamin D level <25 nmol/L at baseline) was associated with 2.1- and 2.6-fold increased risk of low appendicular muscle mass and grip strength, respectively, during a 3-year follow-up period (154). Muscle-specific vitamin D receptor knockout mice have reduced muscle size, impaired motor activity, and abnormal muscle development (155, 156). Vitamin D deficiency also adversely affected skeletal muscle insulin sensitivity, thereby contributing to reduced metabolic flexibility (157). Taken together, these data indicate that vitamin D deficiency and/or its impaired signaling is a critical mediator at the nexus of NAFLD and sarcopenia. Data to support the critical role of vitamin D in directly improving muscle strength and function is provided from several large placebo-controlled randomized controlled trials (RCTs) that demonstrated the effect of vitamin D supplementation on increasing quadriceps strength (158, 159), improving mobility in 6 minute walk (160), jump velocity (161), timed-up-and go (159) tests, and in reducing the incidence of falls (162). Two large meta-analyses which pooled results from 13 RCTs in >60-year-old subjects (163) and another that pooled 17 RCTs in all age groups, including younger subjects (164), suggested that daily vitamin D supplementation (800 IU to 1000 IU per day) was beneficial for muscle strength and balance, especially in those with a baseline serum vitamin D level <25 nmol/L.

## Summary and Future Directions

Multiple factors have been delineated in the pathogenesis of NAFLD/NASH, including immune regulation, lipolysis, leaky gut and bile acid homeostasis, among others. There is close overlap between the pathophysiology of sarcopenia and NASH. This makes it challenging to determine whether sarcopenia is a risk factor for NASH, or if it is a complication of NASH, as the presence of either one may increase the risk for the other. Nonetheless, the current data squarely place skeletal muscle, insulin resistance, and inflammation in the center of the NAFLD/NASH pathogenic cascade. Sarcopenia is widely prevalent and appears to be an effect modifier across the NAFLD spectrum—NAFL, NASH, and fibrosis. Emerging data suggest that it could also potentially be a “causative” factor, although additional studies are needed. Body composition can provide additional insight into the understanding of NAFLD. Interventions that can impact muscle composition, while simultaneously engaging multiple targets/pathways in the muscle-liver axis, would need to be considered to adequately address the complex multifactorial pathogenesis of NAFLD/NASH, and consequently achieve highly effective and durable therapies.

Toward that end, there is a need for deeper understanding of the biology of sarcopenia and its impact on NAFLD. For example, which component of muscle (fat-free muscle volume, or intramuscular fat, or both) impacts more on NAFLD progression and on clinical outcomes? Does myosteatosis still drive NAFLD after adjustment of visceral adiposity? Additional studies are also warranted to better understand various clinical assessment tools, including the prognostic impact of how strength is measured, since handgrip and chair-stand involve two distinct muscle groups, and some of the physical performance test measures are closely related to cardiopulmonary fitness. Dissecting these aspects would be critical to bring forward future individualized recommendations on when to use one test versus the other, which patients differ strongly in hand grip versus chair-stand test, how large is the overlap, and consequently, identify relevant confounders with regard to NAFLD. There is also a need to validate the definitions and cutoff values for muscle mass and function endpoints in clinical trials, which could inform on how to stratify patients in interventional studies, thereby providing better understanding of the magnitude and relevant thresholds of change in those measures associated with clinically meaningful outcomes. Finally, establishing experimental models of sarcopenia and fatty liver disease to elucidate a direct cause-effect relationship between muscle mass and liver lipids would be helpful. Routine measurements of muscle composition and function should also be considered in controlled prospective interventional NASH trials to establish cause-effect relationships in the clinical setting with adjustment for confounding factors such as obesity, physical activity, and inflammation that can influence clinical outcomes.

## Author Contributions

MC conceived, authored the initial draft of the manuscript, and developed the figure concepts. MS, MF, and AS provided further content, added key references, and authored sections of the manuscript. All authors contributed to the article and approved the submitted version.

## Funding

This support was funded by Axcella Health Inc.

## Conflict of Interest

MC is an employee of Axcella Health Inc. and may own stock options in the company. MS has nothing to disclose. MF is an employee of AMRA Medical AB. AS has nothing to disclose for this project. AS is the president of Sanyal Biotechnology and has stock options in Genfit, Akarna, Tiziana, Indalo, Durect, Inversago, and Galmed. He has served as a consultant to Astra Zeneca, Nitto Denko, Conatus, Nimbus, Salix, Tobira, Takeda, Jannsen, Gilead, Terns, Birdrock, Merck, Valeant, Boehringer-Ingelheim, Bristol Myers Squibb, Lilly, Hemoshear, Zafgen, Novartis, Novo Nordisk, Pfizer, Exhalenz, and Genfit. He has been an unpaid consultant to Intercept, Echosens, Immuron, Galectin, Fractyl, Syntlogic, Affimune, Chemomab, Zydus, Nordic Bioscience, Albireo, Prosciento, and Surrozen. His institution has received grant support from Gilead, Salix, Tobira, Bristol Myers, Shire, Intercept, Merck, Astra Zeneca, Malinckrodt, Cumberland, and Novartis. He receives royalties from Elsevier and UptoDate.
